# Does Play After Training Improve a Canine Good Citizenship Skill in Pet Dogs?

**DOI:** 10.3390/ani15101378

**Published:** 2025-05-10

**Authors:** Hannah Salomons, Leah Natalie Ramsaran, Julianna Turner, Brian Hare

**Affiliations:** 1 Evolutionary Anthropology Department, Duke University, Durham, NC 27708, USA; 2 Clinical Sciences Department, College of Veterinary Medicine, Cornell University, Ithaca, NY 14853, USA; 3 Duke Cancer Institute, Duke University, Durham, NC 27708, USA; 4 Center for Cognitive Neuroscience, Duke University, Durham, NC 27708, USA; 5 Psychology and Neuroscience Department, Duke University, Durham, NC 27708, USA

**Keywords:** play, learning, pet dogs, dog training

## Abstract

A major reason people surrender their dogs or fail to adopt them from shelters is due to concerns regarding the management of behavioral issues. This makes effective training of everyday obedience behaviors crucial to dog welfare. Here, we test the playful learning hypothesis to examine whether play after a training session increases a dog’s success in learning two basic Canine Good Citizen behaviors: “sit” and “stay”. In two studies, the dogs experienced brief training sessions and then were assigned to either play or rest. The next day, they returned for another brief training session, and we measured any change in the duration that the dogs were able to hold a “sit” and “stay” from the first day. In Experiment 1, the subjects with low baseline levels of ability improved more after playing than those subjects that simply rested after training. However, Experiment 2, which further controlled for exercise and time spent with trainers, and standardized the measure of success across dogs regardless of their initial ability levels, did not replicate the improved performance in response to play after a training session. Overall, we find limited support for the application of the playful learning hypothesis to everyday training and suggest avenues for future research to determine how play might improve training outcomes for pet dogs.

## Simple Summary

A major reason people surrender their dogs or fail to adopt them from shelters is due to concerns regarding the management of behavioral issues. This makes effective training of everyday obedience behaviors crucial to dog welfare. In this study, we test whether play after a training session increases a dog’s success in learning how to “sit” and “stay”. We conducted two studies, in which half of the dogs rested after their training session, while the other half played, and then we tested how well they performed in training the next day. In the first study, we found some evidence that play after training did help the dogs who were in the early stages of learning these behaviors improve the next day. In the second study, we controlled for the amount of exercise between groups by having the dogs who did not play go for walks during some of this time. In this second study, play did not help improve training. Overall, more research is needed on how play might improve training outcomes for pet dogs.

## 1. Introduction

Dogs are an increasingly important part of family life in many countries and cultures. Family dogs provide meaningful social connections and, in some cases, the most important social bond that many humans have in their lives [1]. This bond has faced a growing challenge as humans have become a predominantly urban-living species over the past few decades. Pet dogs are increasingly expected to show little aggression, no barking, live indoors, and be most active in the evenings and weekends, when their humans are home from work and school. The mismatch between modern expectations and the reality of the needs and capabilities of pet dogs can prevent a human–dog bond or lead to it breaking [2]. Typically, this manifests as a behavior or set of behaviors perceived as problematic for the humans caring for a dog. Dogs identified as having behavior problems often end up on psychotherapeutic drugs, are rehomed, or are surrendered. Behavioral issues also remain a top barrier to the adoption of shelter dogs [3,4]. Identifying positive strategies that can prevent or alter behavior viewed as problematic will quickly improve the lives of pet dogs and represents a welfare priority for researchers.

Socializing and training dogs as puppies and adults remains the main approach to assuring a dog can thrive in their human family. However, not all dogs are well socialized and many pet owners struggle to train their dogs. This means that approaches are needed to help address behavioral issues in adult dogs identified as showing problematic behavior. Research on dog training has a long history and has made significant progress, but has largely focused on laboratory and working dogs [5]. There remains a great need for research on potential training strategies that are simple and effective in improving training outcomes for pet dogs. One promising approach is to consider how the context of training might interact with the ability of adult dogs to learn.

The playful learning hypothesis (PLH) suggests that play facilitates behavioral flexibility and learning [6] and offers an avenue to increase training effectiveness through the incorporation of play during training. A variety of evidence supports the hypothesis that play promotes social and nonsocial learning. Social animals with larger neocortices tend to play more than nonsocial animals [6,7]. Play in highly social species motivates and creates the opportunity for rehearsing critical social skills such as mothering and friendship formation [8,9,10]. Animals deprived of play can also show disrupted neuronal development in the medial prefrontal cortex, which is critical to executive function and learning [11]. As a large-brained social mammal, dogs begin playing before they are weaned and continue using social play throughout their lives to communicate and negotiate relationships with others [12,13]. Even as adults, dogs maintain an unusual level of play, which likely evolved during domestication and promotes the ability of dogs to bond and learn from humans [1,14,15].

The PLH suggests an intimate link between an animal’s ability to play, brain growth, and success in learning socially appropriate behaviors. Based on this link, researchers have begun to explore whether play also can enhance the ability of dogs to learn more arbitrary training tasks. For example, a comparison of puppies who were allowed to play while their mothers completed an odor detection task found that these puppies were five times more successful in odor detection training as adults than puppies who did not have the same opportunity to learn through passive social learning play [16]. A group of Labrador retrievers were also trained with an arbitrary search task in which they had to systematically approach and touch a target object while ignoring a distractor object. The two objects were moved between two hiding locations randomly across trials. After an initial training session, half of the subjects were immediately allowed to play together with a human for half an hour while the other half of the subjects rested during the same period. In the subsequent training session, the play group outperformed the resting group [17]. Eleven dogs were then tested again, one year after the initial playful training, and the individuals who had played after training again performed the best in the same discrimination task [18]. Simply providing a social play period following training enhances the ability of dogs to learn in the short and long term [17,18].

This initial support for the PLH suggests that social play in particular, with humans or other dogs, may enhance the ability of dogs to learn arbitrary tasks. However, Affenzeller and colleagues tested a relatively small and homogeneous sample of dogs, and the task that the dogs were trained on was not relevant to behaviors that could impact rates of pet dog adoption or relinquishment to shelters [19]. The question now becomes whether these discoveries can be replicated when training critical, everyday social skills that dogs need to demonstrate as Canine Good Citizens.

Following Affenzeller and colleagues’ initial demonstration of a link between training outcome and play after a learning session, a test of whether playful learning can improve outcomes with basic obedience training is now needed. This is especially the case for the type of everyday positive behaviors desired by pet dog owners. Here, we test the playful learning hypothesis in two experiments designed to improve outcomes by incorporating play into training of a Canine Good Citizen behavior [20].We focus on training “sit” and “stay”, which are directly relevant to pet and working dogs’ success [20,21]. We also use a shorter play period than Affenzeller and colleagues to simulate a period of play that pet owners would more likely utilize during training. In the first experiment, the PLH predicts that pet dogs (*N* = 31) will learn to sit and stay for longer durations if they play for ten minutes after their initial training instead of resting. It similarly predicts that dogs who start the experiment with high levels of mastery will make greater improvements in their ability to “stay” through real-world distractions if they play, rather than rest, after training. In the second experiment, the PLH predicts dogs (*N* = 36) will again sit and stay longer when their training is followed by a ten-minute play session—even when exercise and the initial level of ability are controlled. 

## 2. Methods

### 2.1. Subjects

For both studies, a selection of pet dog owners from Durham, NC and the surrounding counties were contacted from the Duke Canine Cognition Center’s database of participants who self-enrolled online. Subjects were chosen after filtering the database to exclude dogs with a history of aggression, serious health concerns, and/or who had already passed a Canine Good Citizen test. For Experiment 2, we also filtered out dogs who had already participated in Experiment 1. 

We successfully recruited 40 dogs for each of the two experiments. Before arriving, each dog was randomly pre-assigned to one of two experimental conditions: play or rest. To assure all subjects were comfortable and motivated to learn, subjects were released from the study and dropped from analysis if they showed any signs of discomfort in the testing rooms (e.g., whining or scratching at door to exit) and/or unwillingness to accept a treat from the experimenter. This ultimately yielded thirty-one subjects included in the analysis for Experiment 1 (seventeen in the play condition and fourteen in the rest condition), and thirty-six subjects included in the analysis for Experiment 2 (eighteen in the play condition and eighteen in the rest condition). The included subjects across both experiments ranged in age from 6 months to 11 years, comprised a wide range of purebred and mixed breeds (all breeds are recorded as reported by owners), and had a variety of prior training experiences (Table S1).

### 2.2. Experimental Setup

These experiments took place in three spaces at the Duke Canine Cognition Center in Durham, North Carolina. Participants entered through the waiting room, which was 2.7 m × 3.4 m with linoleum flooring and had cushioned seats and a side table for participant use, a large potted house plant in one corner, and a large TV monitor mounted on the wall, which connected to overhead cameras in the testing room so that owners could watch their dogs’ testing sessions. The waiting room had an interior door that connected to the testing room. The testing room was 6.2 m × 3.2 m, with linoleum flooring, and had a table and chair and some shelving and cabinets on the walls. On the lawn adjacent to the building was the dog play park, which was a fenced 7.6 m by 4.6 m area with an astroturf ground covering. All treats used throughout the experiment were either Zukes Minis, or a preferred treat provided by the owner and cut down to a similar size.

### 2.3. Procedure

All procedures adhered to the regulation set forth by the Duke Institutional Animal Care and Use Committee (Protocol A128-23-06). All subjects were tested individually in the Duke Canine Cognition Center at Duke University. During an acclimation period, subjects were allowed to explore the testing room off-leash for five minutes prior to testing, with owners and experimenters present in the room. During this time, owners were given an overview of the testing protocol and asked to sign a consent form, and experimenters sat on the floor and interacted with the dog by talking in a friendly voice and offering treats and petting them if they approached. After the five-minute acclimation period, each experiment proceeded as described below. The play periods for both experiments took place in the outdoor play park except in the case of poor weather, when they were allowed to play in the testing room (*n*= 1 in Experiment 1, *n* = 3 in Experiment 2). The rest periods for both experiments took place in the waiting room. Owners were present during walks and play or rest sessions, but not during training, unless they were needed for the dog to be motivated and comfortable during testing. Owners were able to watch the training sessions live from the waiting room on a monitor connected to a ceiling camera in the testing room. If a subject made three attempts to go to the door between the testing room and waiting room while whining, barking, and/or scratching at the door, their owner was invited to sit quietly in a chair in a corner of the testing room during training. They were also permitted to praise and pet their dog if the dog approached them, but, otherwise, owners did not participate in the training session. Two experimenters performed all testing. The training experimenter (E1) was responsible for giving “sit” and “stay” commands, increasing stay durations, giving release commands, treating animals when successful and playing with dogs if in the play group. The recording experimenter (E2) was responsible for administering consent forms to owners, recording times and results, and playing with dogs if in the play group. Owners were asked what hand motions they typically used to request their dog “sit” and “stay”. E1 used these hand motions, or if the owner did not report a standard gesture, E1 used an upward-facing flat palm bent at the elbow up towards the chest for “sit”, a flat palm held out toward the dog for “stay”, and “okay!” as the release cue. Water was available *ad libitum* in all locations. 

#### 2.3.1. Experiment 1

**Figure 1:**
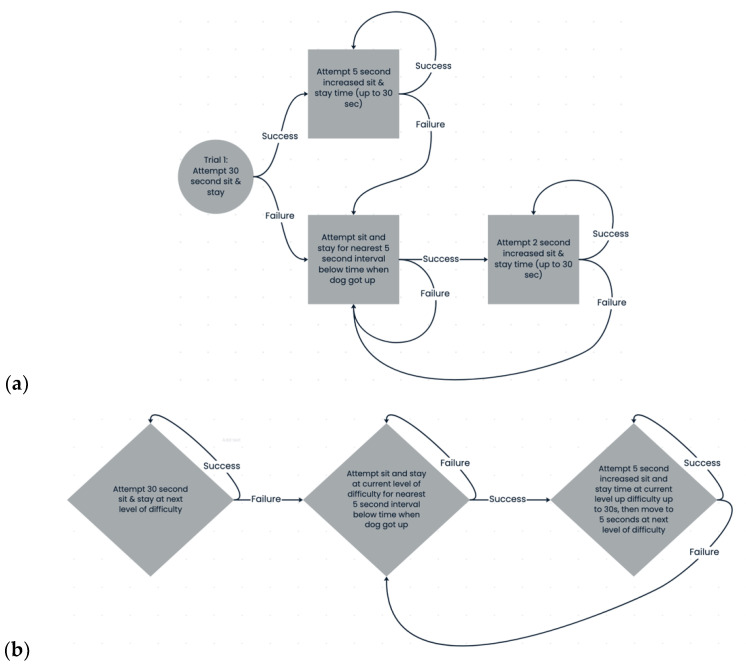
Experiment 1 training flowchart. (**a**) represents the training procedure for dogs who could not yet sit and stay up to 30 s and (**b**) represents the training procedure once dogs were able to stay for thirty seconds and were introduced to increasing distance and distraction.

After the five-minute acclimation period, subjects participated in a five-minute training session, with the goal of introducing or improving on their “sit” and “stay” behaviors. Subjects were administered as many training trials as possible in the five-minute time period. A trial began with E1 standing 0.6 m from the dog and asking the dog to “sit” in an affirmative tone while making eye contact and performing the associated gesture. E1 then asked the dog to “stay” in the same tone while maintaining eye contact and performing the stay gesture. This position would be held until the release cue was given or the dog was no longer in a seated position. E2 used a stopwatch to record the amount of time the dog was able to “stay” for each trial. Timing began when the “stay” command was given. Dogs were given a treat and verbal praise if able to successfully maintain a seated position until released. If a subject stopped sitting (ischium no longer in contact with the ground) before the release, E1 would say “no” in a neutral voice and bring the dog back to starting position. The time the dog would be asked to maintain the “stay” started with 30 s on the first trial—if the dog was not able to maintain the “stay” for the full 30 s in the first trial, the goal for staying in the second trial would be equal to the time they were able to sit in the first trial. After each successful trial, the goal for the time the subject remained in stay increased by five-second intervals. After an unsuccessful trial, the next trial repeated the last successful stay time, and subsequent trials then increased by two-second intervals (Figure 1a). Once the dog could maintain the full 30 s “stay”, training would increase in difficulty level, with the dog staying for 30 s while the experimenter walked away and stood at a distance of two feet, then four feet, then six feet, and finally walked in circles around the dog (Figure 1b). 

After the five-minute training session, all subjects went on a five-minute walk near the test center with E1 and/or E2 and their owner. Subjects then either participated in the play or rest manipulation, depending on which condition they were assigned. The play group was taken outside for ten minutes of play in the Puppy Park with E1, E2, and the owner. E1, E2, and the owner encouraged off-lead play by offering tug of war, fetch, or tag including rope toys, squeaky toys, and tennis balls. Dogs in the rest group were allowed to sit with their owner in the waiting room for ten minutes. Owners were allowed to pet and talk to the dog to ensure the dog did not fall asleep during the rest period. After the ten-minute experimental condition, all subjects went on another five-minute walk with E1 and/or E2 and the owner. At the conclusion of Day 1, owners were not given any particular instructions for the interim before returning for Day 2—if they asked, we instructed them to simply continue their usual routines at home.

**Figure 2:**
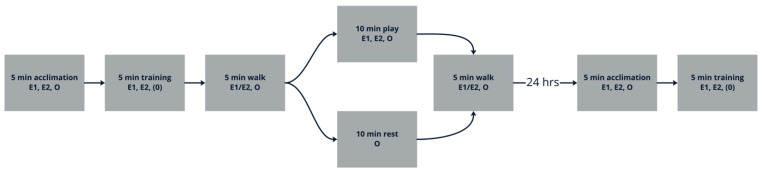
Experiment 1 full procedure flowchart. E1, E2 = experimenters 1 and/or 2 present; O = owner present; (O) = owner only present if dog was distressed when separated.

Owners then brought the subjects back to the DCCC twenty-four hours later. On Day 2, the five-minute acclimation period and five-minute training session were repeated. The training session on Day 2 started by repeating the last successful “stay” time or level from Day 1 and proceeded from there, as described above (Figure 1). See Figure 2 for an illustration of the entire test procedure.

#### 2.3.2. Experiment 2

**Figure 3:**
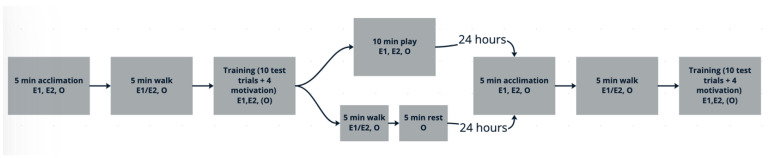
Experiment 2 full procedure flowchart. E1, E2 = experimenters 1 and/or 2 present; O = owner present; (O) = owner only present if dog was distressed when separated.

The procedure was identical to that of Experiment 1 with a few exceptions (see Figure 3). After the five-minute acclimation period, instead of immediately participating in training, all subjects first went on a five-minute walk with E1 and/or E2 and their owner. The subjects then began the training session, but unlike in Experiment 1, there was a set number of trials (10) rather than a set five-minute time limit. Each additional trial increased the duration the subject was asked to “stay” in order to succeed and receive a reward. To ensure subjects remained engaged, four motivation trials were included. These trials only required subjects to “stay” for two seconds in order to succeed and receive a reward. These motivation trials were administered after test trials 2, 4, 6, and 8, so that even if a subject failed to hold their stay for the duration of the test trials, they would potentially maintain motivation to participate after being rewarded in trials of shorter duration. Unlike Experiment 1, subjects were only asked to stay for five seconds on their first test trial. Also, unlike Experiment 1, increasing levels of difficulty were not applied after a subject was able to stay for 30 s—the stay time just continued to increase in five second increments after each successful trial. Thus, in this procedure, a dog who succeeded on all ten trials (i.e., never got up before the release cue) would have stayed 50 s on their last trial before being released. 

Immediately after the training session, the ten-minute experimental condition began. The play condition was the same as described in Experiment 1. In the rest condition, in order to control for exercise and time spent with experimenters, the dogs first went on a five-minute walk with E1 and/or E2 and the owner, and then went to the waiting room to rest, as described in Experiment 1, for the last five minutes. 

Owners then brought the subjects back to DCCC twenty-four hours later. On Day 2, the five-minute acclimation period, five-minute walk, and ten-trial training session were repeated in the same way as described above. The training session on Day 2 started with a warm-up trial requiring the subject to hold a five-second “stay”; then, the first trial of Day 2 was administered using the longest successful “stay” duration achieved on Day 1. Subsequent trials increased in “stay” duration, as described above. See Figure 3 for an illustration of the entire test procedure.

### 2.4. Scoring and Analysis

Subjects in Experiment 1 were sorted into two different groups: dogs with stay durations less than thirty seconds on both days of training (henceforth, the “novice” group, *n* = 21) and dogs that achieved a stay duration of longer than 30 s at least once on either day of training (the “expert” group, *n* = 10). This designation was thus responsive to ability level, but because “expert” status could be achieved on Day 2 after the experimental condition (play or rest) had already been experienced, it was not possible to counterbalance the number of “novices” and “experts” in each condition.

Mean longest “stay” durations were calculated for each day, in each experimental condition, for subjects in the “novice” group in Experiment 1 and for all subjects in Experiment 2. Differences in longest “stay” durations between Days 1 and 2 were then compared between the play condition and rest condition in both experiments using a Welch independent t-test for means with unequal variances. A power analysis performed to ensure that Experiment 2 had sufficient sample size to test for an effect of the size found in Experiment 1 (α = 0.05, power = 0.80).

Subjects meeting the criteria for “expert” dog in Experiment 1 were scored on a scale from 0 to 5 based on the highest level of difficulty they reached during training on each day. They scored 0 points if they could not hold a stay for at least 30 s again; 1 point for staying 30 s; 2 and 3 points for staying 30 s with the experimenter standing 2 and 4 feet, respectively, from the subject; 4 points if they stayed 30 s while the experimenter walked in circles around them; and 5 points if they stayed 30 s while the experimenter circled them while tossing a ball in the air. Mean scores on Day 1 and Day 2 were calculated for dogs in the play condition and rest condition. Mean differences in scores between Days 1 and 2 were also calculated for subjects in each condition. However, the limited number of subjects identified as experts resulted in a sample that was too small and unbalanced across conditions (*n* = 10, 7 in play and 3 in rest condition) to allow for a meaningful quantitative comparison.

Data were analyzed using Excel (Version 16.90) and Tableau (Version 2022.1) software.

## 3. Results

**Figure 4:**
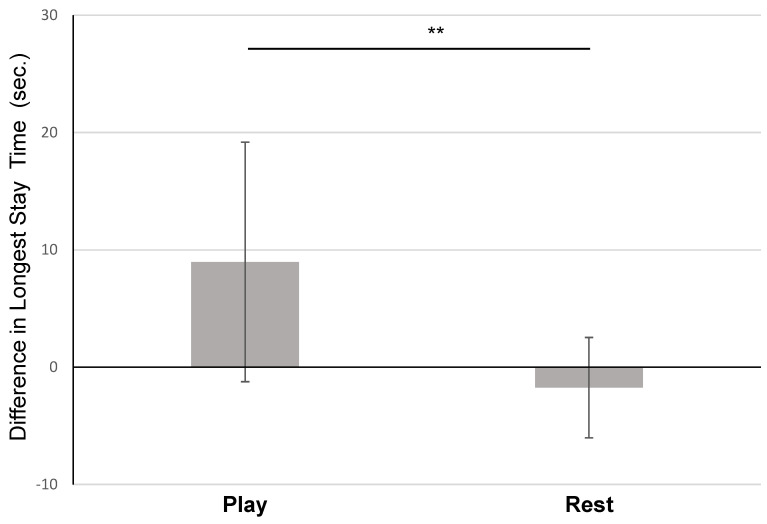
Comparison of dogs who played or rested after training in Experiment 1. Subjects in the play condition improved their a “stay” duration more than those in the rest condition. Difference in longest stay time (Day 2 −Day 1, M ± SD) for dogs in Experiment 1’s novice group, by condition. ** *p* < 0.01.

On Day 1 of Experiment 1 for the “novice” subjects, the mean (±SD) longest “stay” duration in the play condition was 11.93 ± 4.91 s, and the mean longest “stay” duration in the rest condition was 13.91 ± 8.50 s. On Day 2, the mean longest “stay” duration in the play condition increased to 20.91± 7.96 s, whereas the mean longest “stay” duration in the rest condition decreased to 12.16 ± 7.35 s. When comparing the differences in “stay” durations between Day 1 and Day 2, the “novice” subjects in the play condition had a significantly greater increase in duration (*M* = 8.97 s, *SD* = 10.21 s, *n* = 10) than the subjects in the rest condition, with a large effect size (*M =* −1.75 s, *SD* = 4.28 s, *n* = 11, *t*(19) = 3.08, *p* < 0.01, Hedges’ *g* = 1.39) (Figure 4).

On Day 1, for the “expert” subjects, the mean score in the play condition was 1.43 ± 0.55 points, and the mean score in the rest condition was 1.00 ± 1.00 points. On Day 2, the mean score for “expert” subjects in the play condition was 2 ± 1.25 points, and the mean score in the rest condition was 4.00 ± 1.00 points. The mean difference in score between Day 1 and Day 2 for the “expert” subjects in the play condition was 0.29 ± 1.38 points and the mean difference in score in the rest condition was 3 ± 1.73 points. 

**Figure 5:**
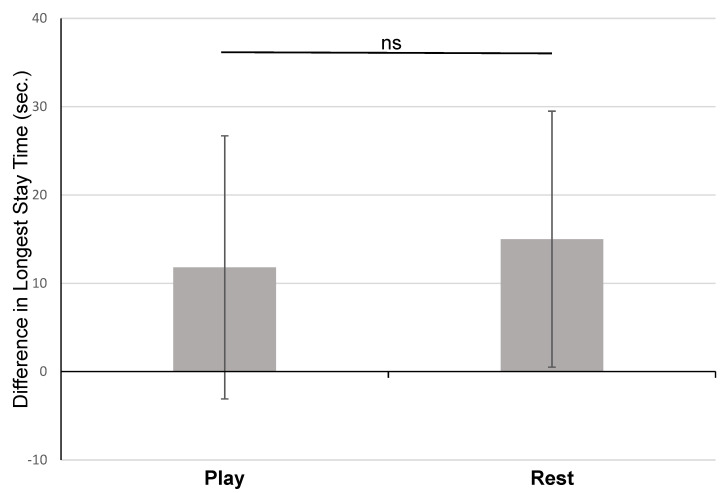
Comparison of dogs who played or rested after training in Experiment 2. Subjects in the play condition did not improve their “stay” duration significantly more than those in the rest condition. Difference in longest stay time (Day 2 −Day 1, M ± SD) for dogs in Experiment 2, by condition.

In Experiment 2, the Day 1 mean longest “stay” duration for subjects in the play condition was 29.2 ± 16.3 s, and the mean longest “stay” duration for subjects in the rest condition was 28.5 ± 15.2 s. On Day 2, the mean longest “stay” duration for the play condition increased to 41.1 ± 23.9 s and the mean longest “stay” duration for the rest condition increased to 43.5 ± 23.7 s. When comparing the differences in “stay” durations between Day 1 and Day 2, there was no significant difference between the subjects in the two conditions (play *M* = 11.8 s, *SD* = 14.9 s, *n* = 18; rest *M* = 15.0, *SD* = 14.5 s, *n* = 18, *t*(34) = −0.636, *p* > 0.05) (Figure 5). A power analysis found that the sample size was more than sufficient to find an effect of the size found in Experiment 1, had there been one (minimum *n* = 10 needed in each condition).

## 4. Discussion

The results of the two experiments only provided limited support to the playful learning hypothesis. Experiment 1’s results partially upheld the prediction that a period of play after a training session would improve the learning of a Canine Good Citizen skill in pet dogs, while Experiment 2’s did not. In Experiment 1, the dogs who were novices (i.e., those dogs unable to hold a stay for 30 s on either day of training) and played after training on Day 1 held their stay significantly longer on Day 2 than the dogs who had rested after training on Day 1. However, the few expert dogs we had (who were capable of holding a stay for 30 s at any point during the experiment) did not improve through play compared to rest—although no quantitative comparison is possible. In Experiment 2, we also did not replicate the pattern from Experiment 1. The subjects’ learning outcomes did not significantly differ between the two conditions—the dogs performed similarly on both days, regardless of whether they had experienced the play or rest condition. 

Our experiments provide a strong test of play’s impact on training for a basic Canine Good Citizenship skill. We chose “sit and stay” as our target training behavior because it is a basic ability that can prevent problems before they happen at home and in public [21]. Many features of our experiments contributed to a strong test of the PLH in this context. First, we were able to almost double the sample size in each experiment from the 16 dogs tested by Affenzeller et al. [17], and provide a more heterogeneous sample of pet dogs, which included dogs from a range of breeds with a variety of life experiences. Second, we shortened the duration of play from the 30 min used in Affenzeller et al. [17] to a ten-minute period that most pet owners could more realistically apply. Third, we introduced additional controls. Unlike Affenzeller et al. [17], in which all dogs were completely naive to the task being trained, in this study, the initial levels of staying ability were controlled for by randomly assigning the experimental condition. The average stay times on Day 1 were similar between conditions in both studies, confirming that subjects across conditions started at a similar level of average mastery prior to their randomly assigned intervention. The average stay times at the completion of Day 1 training were much higher in Experiment 2 than they were on the first day of Experiment 1. This suggests that our changes in the training protocol in Experiment 2 increased the training effectiveness. It is likely that starting with a 5 s “warm-up” trial, where most dogs had a chance to be successful on the first trial, rather than beginning with an attempt at a 30 s stay (where many dogs failed), increased motivation and helped the dogs to understand the behavior we were asking of them. We also introduced a control for exercise. In Experiment 1, the dogs in both conditions went for walks before training and after the experimental intervention on Day 1, so they all experienced the same baseline level of exercise. In Experiment 2, the level of exercise was further equalized by the dogs in the rest group going for an additional walk during half the intervention time period. Finally, we controlled for time spent with the trainer in Experiment 2 by having the trainer join dogs in the rest condition on this additional walk, since the trainer was with the dogs during the play period.

While our experiments provide a strong test of the PLH, our results differ from those of previous experiments. The results here do not provide robust evidence that play after training improves learning outcomes; therefore, if play does have an effect, it may be specific to certain contexts or types of learning. The behaviors trained in the current research may recruit different cognitive skills than the object discrimination task used by Affenzeller et al. [17]. Sitting and staying requires inhibitory control. Success requires an understanding of the request and inhibition of the desire to do some other behavior. It is possible that play after training helps dogs synthesize or encode new information, like that introduced in an object discrimination task, but it does not help with requests that demand inhibitory control. This idea is first suggested by the lack of qualitative improvement in the expert dogs from Experiment 1 and supported by the rapid learning in Experiment 2. The dogs from Experiment 2 already reached a mean of almost 30 s for holding their stay on Day 1, and then there was no difference between play and rest on Day 2. Thus, it is possible that play may help in learning a new concept, but does not enhance inhibitory control that is needed to maintain a behavior like staying for extended periods.

Another factor which may have impacted the results was exercise. It is well established that exercise promotes learning and memory in humans and rodents [22,23,24], and even a single bout of exercise can have an effect [25]. It is possible that the improvement of the dogs in the play condition found in Experiment 1 was actually due to a difference in exercise between the two conditions. In Experiment 2, where exercise was better controlled, no significant effect of play was found. This interpretation is further supported by the fact that the dogs in Affenzeller et al. [17] played (i.e., exercised) for a much longer time period than those in the current study (30 min compared to 10 min) and the heart rates of the dogs in the play condition were measurably elevated compared to those of the dogs in the rest condition. However, in Experiment 1 of the current research, the subjects in the rest condition still did perform some exercise (albeit less than those in the play condition), so further investigation may be warranted. If just an additional ten minutes of exercise, as the dogs in Experiment 1 experienced, really did cause the training improvement, this would be an exciting and easily implementable finding for pet dog owners. Future research can compare play with brisk walking, instead of rest, after training to disentangle the role of these variables.

A final potential explanation for our results is that play is important in strengthening the bond between the trainer and the dog, and, therefore, indirectly improves learning by improving the dog’s motivation to participate and increasing attention to the trainer during training sessions. This would be supported by the finding that when the time spent with the trainer was controlled between the play and rest group in Experiment 2, there was no difference in their Day 2 outcomes. Furthermore, in a recent longitudinal study of cognitive development [26], puppies who were raised with and spent more time playing with other puppies and a larger number of unfamiliar people during rearing did not significantly differ in their success in a battery of cognitive tasks from those raised in a typical home setting. This suggests that more varied play partners may not impact learning and cognition. However, while these puppies played with a wider variety of social partners, they did not spend more time playing with the specific individuals who tested them on these tasks. It may be that the impact of play will be observed when play is performed with a trainer with whom the dog is bonded.

It is also possible that a combination of training task, exercise, and dog-trainer relationship all contributed to the difference in the effect of play found between this study and those of Affenzeller et al. [17,18]. Thus, although we did not see a robust and consistent effect of play after training on our target behavior, it remains important to continue exploring the role play has in improving training techniques for pet dogs. It remains a priority for future research to systematically manipulate the factors potentially contributing to the training outcomes we observed and further test the impact of play on basic good citizenship behaviors in dogs.

One first step would be to separate out the processes of learning a new concept from increasing inhibitory control. This could be accomplished by repeating Experiment 2 with dogs who have yet to be introduced to requests to sit and stay (either young puppies, or shelter dogs who have no response to those commands). In the current study, an improvement from 0 s to 5 s carried the same weight as an improvement from 30 s to 35 s, but it is likely that these are cognitively different changes. Additionally, subjects had a wide variety of training histories reported by their owners, from no training, to home training of all kinds, to puppy/socialization classes, to competing in dog sports. We were therefore unable to make any kind of quantitative evaluation of the effect of prior training in this study. In the future, a subject pool of dogs with zero level of mastery coming in (such as young puppies, or those whose owners report no “stay” training) would make comparisons between the play and rest conditions more readily interpretable.

Another future direction includes additional control(s) for exercise levels, using heart rate monitors to ensure that dogs in both conditions are similarly active. In the current study, this was not systematically controlled. The dogs in the play condition engaged in varying levels of exercise depending on their preferred type of play (fetch involves more running than tug, for example).

Additionally, to further parse out the interaction between play and a subject’s relationship with the trainer, the dogs’ training outcomes can be compared when they are trained by someone they are bonded with versus someone unfamiliar. Similarly, since a number of the recruited subjects (~16%) did not complete the study due to discomfort in the testing room and/or unwillingness to accept a treat from the unfamiliar experimenter, our findings may not necessarily be applicable to dogs who are nervous in new situations. Future work taking place in dogs’ familiar home environments would be more generalizable.

Finally, since many of the studies on playful learning with children incorporate play into the actual teaching [27,28], future research can be performed on playing *during* training with dogs, rather than after. This will help test if play while training dogs might also show the effect seen in children, and if it is more robust to the varying factors described above.

## 5. Conclusions

Limited support was found for the playful learning hypothesis in these studies. Understanding if, and how, play can improve everyday training outcomes has potential for increasing companion dog welfare. Hopefully, future research will improve dog welfare by identifying simple training strategies, like play during training, or extended play that raises the heart rate after training, which could increase the proportion of dogs who can show good citizenship in public, succeed as working dogs, and be ready for adoption from shelters.

## Data Availability

The original contributions presented in this study are included in the Supplementary Materials. Further inquiries can be directed to the corresponding author.

## Supplementary Materials

The following supporting information can be downloaded at https://www.mdpi.com/article/10.3390/ani15101378/s1: Table S1: Dataset.
